# Impact of Coffee/Green Tea/Soft Drink Consumption on the Risk of Hyperuricemia: A Cross-Sectional Study

**DOI:** 10.3390/ijerph18147299

**Published:** 2021-07-08

**Authors:** Joong Seob Lee, Tae Jun Kim, Sung Kwang Hong, Chanyang Min, Dae Myoung Yoo, Jee Hye Wee, Hyo Geun Choi

**Affiliations:** 1 Department of Otorhinolaryngology-Head and Neck Surgery, Hallym University College of Medicine, Anyang 14068, Korea; apniosio@naver.com (J.S.L.); yeramii@hanmail.net (S.K.H.); weejh07@gmail.com (J.H.W.); 2 Department of Medicine, Samsung Medical Center, Sungkyunkwan University School of Medicine, Seoul 06351, Korea; taejunk91@gmail.com; 3 Hallym Data Science Laboratory, Hallym University College of Medicine, Anyang 14068, Korea; joicemin@naver.com (C.M.); ydm1285@naver.com (D.M.Y.)

**Keywords:** hyperuricemia, soft drink intake, nutrition, cross-sectional study

## Abstract

This cross-sectional study aimed to investigate the association between hyperuricemia and the frequency of coffee, tea, and soft drink consumption, based on data from the Korean Genome and Epidemiology Study (KoGES) (2004–2016). We used the KoGES health examinee data, obtained from urban residents aged ≥ 40 years. Information on the participants’ medical history, nutrition (total calorie, protein, fat, and carbohydrate intake), frequency of alcohol consumption, smoking status, household income, and frequency of coffee/green tea/soft drink intake was collected. A logistic regression model was used to analyze the data. Subgroup analyses were performed according to the participant’s age and sex. Among 173,209 participants, there were 11,750 and 156,002 individuals with hyperuricemia and non-hyperuricemia controls, respectively. In an adjusted model, frequent coffee and green tea consumption did not increase the risk of hyperuricemia, compared to the “no intake” reference group. However, an adjusted odds ratio of hyperuricemia was 1.23 (95% confidence interval, 1.11–1.35, *p* < 0.001) for participants who reported consuming soft drinks ≥ 3 times per day, compared to the respective “no drink” reference group. Even after adjusting for nutritional and sociodemographic factors, frequent soft drink intake was associated with an increased risk of hyperuricemia. Meanwhile, neither coffee nor green tea intake was associated with an increased risk of hyperuricemia.

## 1. Introduction

Hyperuricemia is the main risk factor for gout and plays a major role in its pathogenesis [1]. Gout is a common form of inflammatory arthritis, characterized by sudden and severe pain, and swelling of the affected joint [2], impairing patients’ quality of life [3]. In addition to being a risk factor for inflammatory arthritis, elevated levels of serum uric acid are associated with an increased risk of cardiovascular disorders [4]. According to the National Health and Nutrition Examination Survey, 21% of Northern America’s adult population aged > 20 years are affected by hyperuricemia [5]. In Korea, the age-standardized prevalence of hyperuricemia has been estimated at 11.4% [6]. Hyperuricemia is prevalent worldwide, and the number of people affected by it has increased over the past two decades [7].

Various factors affect serum uric acid concentration, including dietary habits, and the diagnosis of obesity and metabolic syndrome [8]. Studies have shown that a high intake of purine-rich food (for example bacon, meat, and pork) and beer can increase the risk of hyperuricemia [9,10]. Meanwhile, the intake of dairy products (for example milk and yogurt) has been shown to reduce serum uric acid level [10,11]. Low-calorie intake and a diet that involves restricted carbohydrate consumption can also induce hyperuricemia [12,13]. This evidence suggests that achieving a nutritional balance (including in total calorie, protein, fat, and carbohydrate intake) is paramount before commencing further investigations into the causes of hyperuricemia.

Tea and coffee are among the most commonly consumed beverage types worldwide [14,15], while the consumption of soft drinks continues to increase [16]. Recent studies have reported an association between serum uric acid level and the intake of coffee, tea, and soft drinks [17,18,19]. It has been hypothesized that the fructose present in soft drinks may increase the risk of hyperuricemia [18]; however, the exact mechanism through which these beverages may induce hyperuricemia remains unclear.

Thus, in the present study, we aimed to investigate the association between hyperuricemia and the frequency of coffee, tea, and soft drink consumption. We adjusted the nutritional and sociodemographic factors in a cross-sectional design, using population-based data. The secondary aim of this study was to estimate the risk of hyperuricemia, stratified by age and sex.

## 2. Materials and Methods

The present study protocol was approved by the ethics committee of Hallym University (No. 2019-02-020); the requirement for informed consent was waived. This cross-sectional study was based on the Korean Genome and Epidemiology Study (KoGES) dataset (2004–2016), details of which have been previously reported [20]. From the KoGES Consortium, we extracted the health examinee (HEXA) data, obtained from urban residents aged ≥ 40 years. Baseline data and follow-up data were acquired during 2004–2013 and 2012–2016, respectively.

### 2.1. Participant Selection

**Figure 1:**
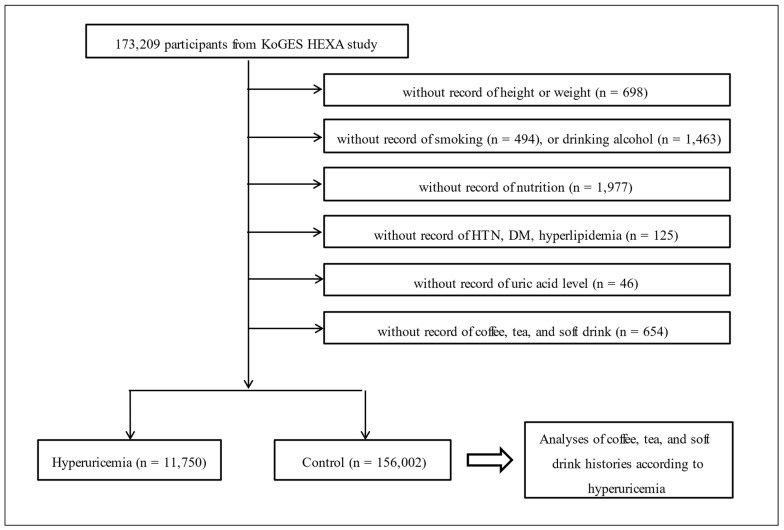
The selection of the study’s participants. A total of 11,750 participants with hyperuricemia and 156,002 participants with non-hyperuricemia controls were analyzed.

Of 173,209 participants, we excluded participants with missing data on height or weight (*n* = 698); smoking history (*n* = 494); alcohol drinking habits (*n* = 1463); nutritional status (*n* = 1977); history of hypertension, diabetes mellitus, or hyperlipidemia (*n* = 125); serum uric acid levels (*n* = 46); coffee, green tea, or soft drink intake (*n* = 654). The final sample included 11,750 and 156,002 participants with hyperuricemia and non-hyperuricemia controls, respectively (Figure 1). The frequency of coffee, green tea, and soft drink consumption was investigated.

### 2.2. Survey

Participant history of hypertension, diabetes mellitus, and hyperlipidemia was acquired during face-to-face interviews, conducted by expert interviewers. Although hyperuricemia does not have a consensus definition based on the level of serum uric acid, the present study defined hyperuricemia as serum uric acid levels of >7.0 mg/dL in men and of >6.0 mg/dL in women, based on the previous literature [5,21]. Body mass index (BMI) was defined as weight over height-squared (kg/m^2^), calculated based on health checkup data. Smoking history was classified as non-smoker (<100 cigarettes during lifetime), past smoker (ceased smoking > 1 year before study enrollment), and current smoker. Alcohol intake was categorized as nondrinker, past drinker, and current drinker. Nutritional intake (total calories [kcal/day], protein [g/day], fat [g/day], and carbohydrate [g/day]) was assessed based on a food-frequency questionnaire, which has been previously described and validated [22]. Household income was categorized as follows: undisclosed low income (up to $2000 per month), middle income ($2000–3999 per month), and high income (≥$4000 per month).

The frequency of coffee, green tea, and soft drink intake was classified as “never,” 1 time per month, 2–3 times per month, 1–2 times per week, 3–4 times per week, 5–6 times per week, 1–2 times per day, 3–4 time per day, and ≥5 times per day. Given the distribution of these categories in the study sample, we reclassified them into four groups as follows: never, 1 time a month through 6 times a week, 1–2 times a day, and ≥3 times a day.

### 2.3. Statistical Analyses

Chi-square tests were used to compare the rate of sex, income group, smoking, drinking and alcohol consumption, and the frequency and amount of coffee/green tea/soft drink consumption. To compare the age, BMI, and nutritional intake, an independent *t*-test was applied.

To investigate the impact of coffee, green tea, and soft drink intake on the risk of hyperuricemia, we computed three models: a crude, model 1 (adjusted for age, sex, BMI, income, smoking, past medical histories, alcohol consumption, and nutritional intake [total calories, protein, fat, carbohydrate intake]), and model 2 (fully-adjusted model, including variables from model l as well as independent variables, such as the frequency of coffee, green tea, and soft drink consumption). Effect estimates were expressed as odds ratios (OR).

In age-based subgroup analyses, the data were dichotomized at the median value (≤52 vs. ≥53 years).

Two-tailed analyses were performed, and *p*-values of <0.05 were considered indicative of statistical significance. Statistical analysis was performed using SPSS v. 24.0 (IBM Corp. IBM SPSS Statistics for Windows, Version 24.0, Armonk, NY, USA).

## 3. Results

Participant characteristics are presented in Table 1. The participants in the hyperuricemia group were older than the participants in the control group, and their BMI was higher than that of the control group. The proportion of men was significantly higher in the hyperuricemia group than in the control group. There were between-group differences in household income, smoking status, and alcohol consumption. The rate of hypertension, diabetes mellitus, and hyperlipidemia was significantly higher in the hyperuricemia group than in the control group. Nutritional status, including total calorie, protein, fat, and carbohydrate intake was significantly higher in the hyperuricemia group than in the control group. There were significant between-group differences in the frequency of coffee, tea, and soft drink intake.

Table 2 presents the crude and adjusted ORs of hyperuricemia, based on the frequency of coffee, green tea, and soft drink intake. In model 1, the adjusted OR of hyperuricemia was 1.09 (95% confidence interval [CI], 1.02–1.16, *p* = 0.008) for participants who consumed soft drinks 1–2 times per day; it was 1.25 (95% CI, 1.13–1.37, *p* < 0.001) for participants who consumed soft drinks ≥ 3 times per day compared to the reference group of “never” consumption (Table 2). In model 2, the adjusted OR of hyperuricemia was 1.07 (95% CI, 1.01–1.14, *p* = 0.028) in participants who consumed soft drinks 1–2 times per day; it was 1.23 (95% CI, 1.11–1.35, *p* < 0.001) in participants who consumed soft drinks ≥ 3 times per day, compared to the reference group of “never”.

**Figure 2:**
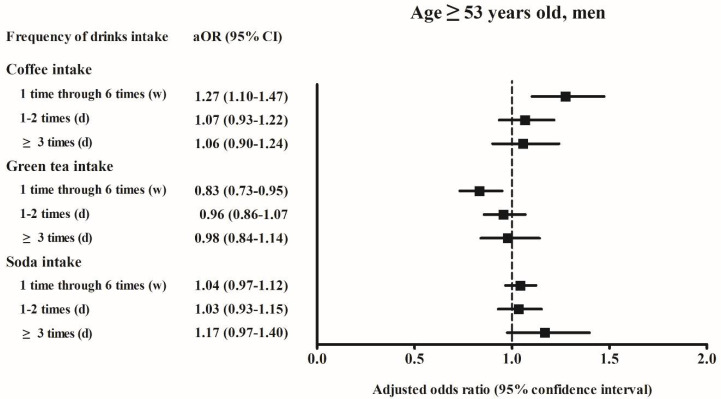
The adjusted odds ratio of the hyperuricemia according to the frequency of coffee/green tea/soda (soft drink) intake in the age ≥ 53 years old men. Fully-adjusted model (age, BMI, income, past medical histories, smoking, alcohol consumption, nutritional intake, frequency of coffee, green tea, and soft drink intake) was used.

**Figure 3:**
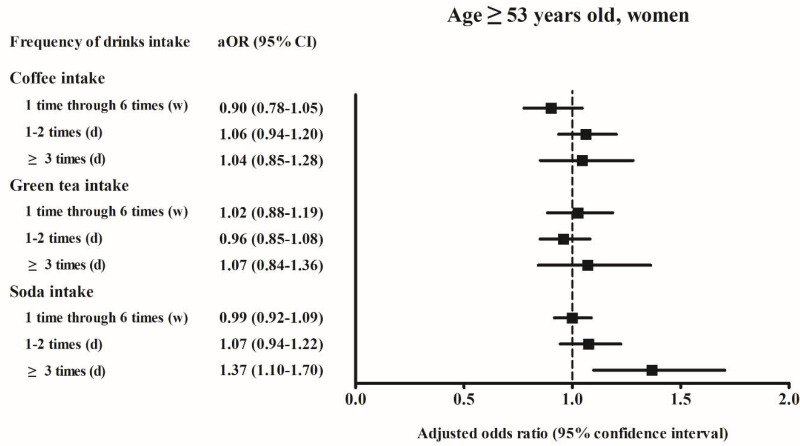
The adjusted odds ratio of the hyperuricemia according to the frequency of coffee/green tea/soda (soft drink) intake in the age ≥ 53 years old women. Fully-adjusted model (age, BMI, income, past medical histories, smoking, alcohol consumption, nutritional intake, frequency of coffee, green tea, and soft drink intake) was used.

**Figure 4:**
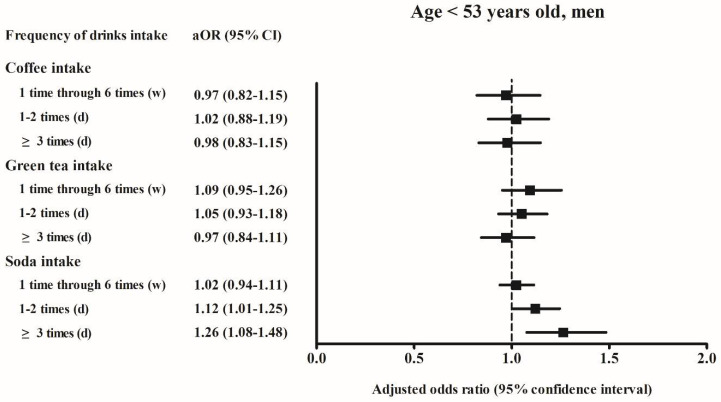
The adjusted odds ratio of the hyperuricemia according to the frequency of coffee/green tea/soda (soft drink) intake in the age < 53 years-old men. Fully-adjusted model (age, BMI, income, past medical histories, smoking, alcohol consumption, nutritional intake, frequency of coffee, green tea, and soft drink intake) was used.

**Figure 5:**
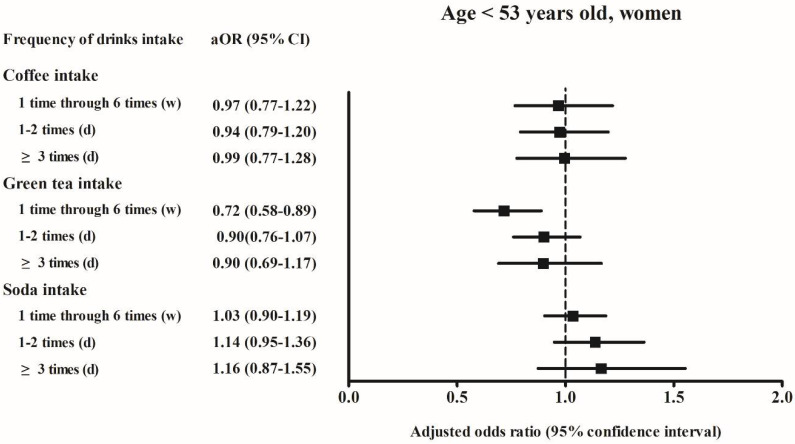
The adjusted odds ratio of the hyperuricemia according to the frequency of coffee/green tea/soda (soft drink) intake in the age < 53 years old women. Fully-adjusted model (age, BMI, income, past medical histories, smoking, alcohol consumption, nutritional intake, frequency of coffee, green tea, and soft drink intake) was used.

Among men aged < 53 years who consumed soft drinks ≥ 3 times per day, the adjusted OR of hyperuricemia was 1.26 (95% CI, 1.08–1.48, *p* = 0.004) compared to the reference group of “never”. Among women aged ≥ 53 years who consumed soft drinks ≥ 3 times per day, the adjusted OR of hyperuricemia was 1.37 (95% CI, 1.10–1.70, *p* = 0.005) compared to the reference group of “never” (Figure 2, Figure 3, Figure 4 and Figure 5).

## 4. Discussion

In the present study, participants who reported frequent consumption of soft drinks were at increased risk of hyperuricemia compared to their counterparts who declared “never” in their consumption; this association remained after adjustments for sociodemographic and nutritional factors. Adjusting in relation to nutritional factors is paramount in this type of study, as nutrition can affect the level of serum uric acid. Previous studies on this topic have reported findings based on similar adjustments [18,23]. In their reports, the impact of total calorie, protein, alcohol, meat, seafood, and dairy product intake were considered [18,23]. However, our study involved adjustments for the intake of all three major nutrients (carbohydrate, fat, and protein), reported in grams per day, which is rare; nevertheless, this approach ensures the accuracy of analysis and ease of comparison between the present findings and those of previous studies.

Choi et al. reported in their prospective study that an intake of sugar-sweetened soft drinks was strongly related to the elevated risk of gout in men [24]. In their report, the relative risk of gout was 1.85 (95% CI, 1.08–3.16) in men who consumed sugar-sweetened soft drinks > 2 times per day, compared to men who consumed them < 1 time per month. Interestingly, intake of “diet” soft drinks (for example, low-calorie or caffeine-free Coca-Cola, or other types of low-calorie beverages) did not affect the risk of gout in that study. Another report from the Korean population demonstrated that increased soft drink intake was associated with the increased risk of hyperuricemia in men [25]. In their report, the adjusted OR of hyperuricemia was 1.35 (95% CI, 1.07–1.71) in the higher intake group (Q5), compared to the lower intake group (Q1–Q3). Serum uric acid level can be affected by the boosted uric acid production via fructose metabolism. Throughout the fructose metabolism in the cell after fructose uptake, adenosine triphosphate (ATP) depletion induces adenosine monophosphate (AMP) accumulation and AMP deaminase stimulation, leading to the production of uric acid in the cell [26].

In our study, men aged < 53 years and women aged ≥ 53 years who consumed soft drinks ≥ 3 times per day were at increased risk of hyperuricemia (Figure 2 and Figure 3). A previous study based on the national health and nutrition examination survey data reported that the increase in serum uric acid levels related to soft drink consumption was significantly larger among men than it was among women [18]. In an animal study based on rats, female sex hormones have shown to have a protective effect against hyperinsulinemia associated with fructose consumption [27]. In a separate study, hyperinsulinemia has been shown to decrease the rate of renal excretion of uric acid and correlate with elevated levels of serum uric acid [28]. In our study, women aged ≥ 53 years might have had lower estrogen levels than those observed among women aged < 53 years. The present study findings support the hypothesis that estrogen may have a protective effect against the development of hyperuricemia.

Although a previous study reported that men aged > 50 years with dietary fructose intake are at increased risk of hyperuricemia [29], the reason for the significant association between soft drink consumption and hyperuricemia risk observed in men aged < 53 years in the present study remains unclear. Potentially confounding factors, such as lack of exercise routine in the middle-aged men [30] and emotional stress in the workplace [31], may affect the final results in a relatively young male group. Therefore, further studies are required to elucidate these associations.

A few studies have reported on the association between coffee consumption and serum uric acid levels. However, their findings were inconsistent. One recent meta-analysis demonstrated a lack of evidence for an association between coffee consumption and reduced serum uric acid levels [17]. Nevertheless, a previous study has reported on the protective effect of coffee consumption against hyperuricemia risk [32]. In the present study, frequent coffee intake was not significantly associated with hyperuricemia risk (Table 2). However, further studies are required to clarify this association.

In the present study, green tea consumption was not significantly associated with hyperuricemia risk. Previous studies in animals have shown that ingredients present in green tea may decrease serum uric acid levels [33,34]. Although a similar finding might have been expected in humans, previous studies have been inconclusive [35,36]. Consistent with our results, a recent meta-analysis has demonstrated lack of evidence that tea consumption is associated with serum uric acid level [19]. Although our results support this overall finding, further studies are needed before conclusions can be drawn.

The present study has several limitations. Firstly, we did not evaluate the exact amount of coffee, green tea, and soft drinks consumed, as the data were collected with a questionnaire that used frequency categories rather than volume estimates. Secondly, the influence of medication use on serum uric acid levels was not evaluated. Many types of medication, including anti-tubercular drugs, low-dose aspirin, chemotherapy drugs, and diuretics have been reported to increase the level of serum uric acid [37]. Aspirin is widely available in Korea without a prescription, therefore the amount of aspirin consumed cannot be estimated based on medical records.

Notwithstanding these weaknesses, this study has several strengths. Firstly, this study included a large sample size. The HEXA dataset included a large number of participants with hyperuricemia. To the best of our knowledge, this is the largest study of its kind done to-date. Secondly, this study accounted for candidate confounders, including nutritional intake and alcohol consumption. In addition, we applied model 2 (in Table 2) to our analysis in order to minimize the mutual influences of drinking habits. These aspects of the study design helped to ensure the validity of the presented findings.

## 5. Conclusions

This study has shown that frequent soft drink consumption is associated with an increased risk of hyperuricemia. Subgroup analysis revealed that this effect particularly affects men aged < 53 years and women aged ≥ 53 years. Further studies are required to fully elucidate the association between soft drink intake and the risk of hyperuricemia, including the underlying mechanism.

## Figures and Tables

**Table 1:** General Characteristics of Participants.

Characteristics	Total Participants			*p*-Value
	Total	Hyperuricemia	Control	
Total (*n*)	167,752 (100.0)	11,750 (100.0)	156,002 (100.0)	
Age (mean, SD, y)	53.1 (8.3)	54.9 (8.7)	53.0 (8.3)	<0.001 *
Sex (*n*, %)				<0.001 *
Men	57,596 (34.3)	7757 (66.0)	49,839 (31.9)	
Women	110,156 (65.7)	3993 (34.0)	106,163 (68.1)	
BMI (mean, SD, kg/m^2^)	23.9 (2.9)	25.5 (3.0)	23.8 (2.9)	<0.001 *
Income (*n*, %)				<0.001 *
Missing, no response	26,423 (15.8)	2069 (17.6)	24,354 (15.6)	
Lowest	45,908 (27.4)	3231 (27.5)	42,677 (27.4)	
Middle	60,465 (36.0)	4014 (34.2)	56,451 (36.2)	
Highest	34,956 (20.8)	2436 (20.7)	32,520 (20.8)	
Smoking status (*n*, %)				<0.001 *
Nonsmoker	122,197 (72.8)	5755 (49.0)	116,442 (74.6)	
Past smoker	24,651 (14.7)	3499 (29.8)	21,152 (13.6)	
Current smoker	20,904 (12.5)	2496 (21.2)	18,408 (11.8)	
Alcohol consumption (*n*, %)				<0.001 *
Non drinker	85,200 (50.8)	3988 (33.9)	81,212 (52.1)	
Past drinker	6607 (3.9)	732 (6.2)	5875 (3.8)	
Current drinker	75,945 (45.3)	7030 (59.8)	68,915 (44.2)	
Hypertension	37,362 (22.3)	4757 (40.5)	32,605 (20.9)	<0.001 *
Diabetes mellitus	13,243 (7.9)	1237 (10.5)	12,006 (7.7)	<0.001 *
Hyperlipidemia	22,122 (13.2)	2027 (17.3)	20,095 (12.9)	<0.001 *
Nutritional intake				
Total calories (kcal/d)	1758.6 (590.1)	1786.4 (595.9)	1756.5 (589.6)	<0.001 *
Protein (g/d)	59.9 (27.1)	61.1 (27.6)	59.8 (27.1)	<0.001 *
Fat (g/d)	28.2 (18.7)	29.0 (19.0)	28.1 (18.6)	<0.001 *
Carbohydrate (g/d)	312.2 (96.5)	315.5 (96.6)	312.0 (96.5)	<0.001 *
Frequency of coffee				<0.001 *
No drink	71,674 (16.7)	1744 (14.8)	26,327 (16.9)	
1 time (m) though 6 times (w)	27,483 (21.6)	2438 (20.7)	33,745 (21.6)	
1–2 times (d)	47,595 (42.2)	4877 (41.5)	65,876 (42.2)	
≥3 times (d)	21,000 (19.5)	2691 (22.9)	30,054 (19.3)	
Frequency of tea				<0.001 *
No drink	71,674 (42.7)	4680 (39.8)	66,994 (42.9)	
1 time (m) though 6 times (w)	27,483 (16.4)	1846 (15.7)	25,637 (16.4)	
1–2 times (d)	47,595 (28.4)	3391 (28.9)	44,204 (28.3)	
≥3 times (d)	21,000 (12.5)	1833 (15.6)	19,167 (12.3)	
Frequency of soda drink				<0.001 *
No drink	66,392 (39.6)	4534 (38.6)	61,858 (39.7)	
1 time (m) though 6 times (w)	72,468 (43.2)	4921 (41.9)	67,547 (43.3)	
1–2 times (d)	22,641 (13.5)	1744 (14.8)	20,897 (13.4)	
≥3 times (d)	6251 (3.7)	551 (4.7)	5700 (3.7)	

d: day; w: week; m: month; * Independent *t*-test or Chi-square test. Significance at *p* < 0.05.

**Table 2:** Crude and adjusted odd ratios (95% confidence interval) of coffee, green tea, and soft drink intake (frequency) for hyperuricemia.

Characteristics	Odd Ratios for Hyperuricemia					
	Crude	*p*-Value	Model 1 ^†^	*p*-Value	Model 2 ^‡^	*p*-Value
Total participants (*n* = 167,752)						
Coffee						
No drink	1.00		1.00		1.00	
1 time (m) though 6 times (w)	1.09 (1.02–1.16)	0.007 *	0.95 (0.89–1.02)	0.154	0.99 (0.91–1.07)	0.788
1–2 times (d)	1.12 (1.06–1.18)	<0.001 *	0.97 (0.91–1.03)	0.279	0.98 (0.91–1.05)	0.509
≥3 times (d)	1.35 (1.27–1.44)	<0.001 *	0.95 (0.88–1.01)	0.105	0.95 (0.87–1.04)	0.251
Green tea						
No drink	1.00		1.00		1.00	
1 time (m) though 6 times (w)	1.03 (0.98–1.09)	0.287	0.95 (0.89–1.00)	0.063	0.95 (0.88–1.02)	0.161
1–2 times (d)	1.10 (1.05–1.15)	<0.001 *	0.98 (0.93–1.03)	0.407	0.99 (0.93–1.05)	0.665
≥3 times (d)	1.37 (1.29–1.45)	<0.001 *	0.96 (0.90–1.02)	0.204	0.99 (0.91–1.08)	0.809
Soft drink						
No drink	1.00		1.00		1.00	
1 time (m) though 6 times (w)	0.99 (0.95–1.04)	0.776	1.02 (0.98–1.06)	0.412	1.01 (0.97–1.06)	0.615
1–2 times (d)	1.14 (1.08–1.21)	<0.001 *	1.09 (1.02–1.16)	0.008 *	1.07 (1.01–1.14)	0.028 *
≥3 times (d)	1.32 (1.20–1.45)	<0.001 *	1.25 (1.13–1.37)	<0.001 *	1.23 (1.11–1.35)	<0.001 *

* Logistic regression model, Significance at *p* < 0.05; ^†^ Model 1 was adjusted for age, sex, BMI, income, past medical histories, smoking, alcohol consumption, and nutritional intake; ^‡^ Model 2 was adjusted for model 1 plus frequency of coffee, green tea, and soft drinks.

## Data Availability

Restrictions apply to the availability of these data. Data was obtained from Korean Genome and Epidemiologiy Study [KoGES] and are available at [www.kdca.go.kr] (accessed on 23 May 2021), with the permission of [KoGES].
